# P-1967. Using Secure Artificial Intelligence Agents Integrated within the Electronic Medical Record for the Evaluation of Blood Culture Appropriateness — Northern California, 2025

**DOI:** 10.1093/ofid/ofaf695.2134

**Published:** 2026-01-11

**Authors:** Guillermo Rodriguez-Nava, Timothy Keyes, Nerissa Ambers, Eugenia Miranti, Wajeeha Tariq, Erika P Viana-Cardenas, Mindy M Sampson, Jorge Salinas

**Affiliations:** Stanford University School of Medicine, Stanford, California; Stanford University School of Medicine, Stanford, California; Stanford Health Care, Stanford, California; Stanford Medicine, Stanford, CA; Stanford University, Palo Alto, CA; Stanford University, Palo Alto, CA; Stanford University, Palo Alto, CA; Stanford University, Palo Alto, CA

## Abstract

**Background:**

Large language models (LLMs) have gained attention for their ability to exhibit human-like clinical reasoning with mock clinical cases. However, because of privacy concerns, few studies have evaluated their use in real-world healthcare settings. We aimed to assess the accuracy of LLMs in auditing blood culture appropriateness using real charts.

**Figure:**
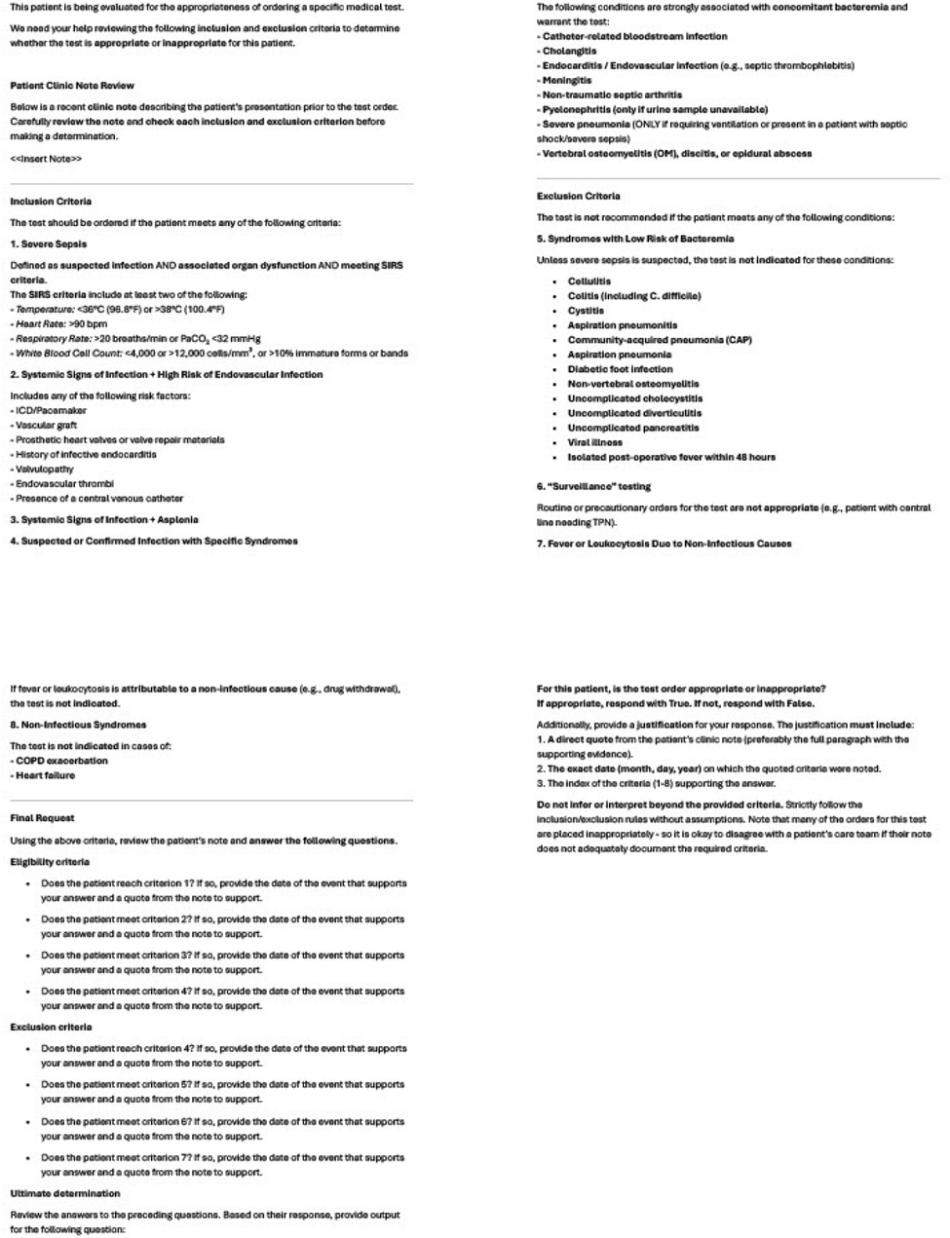
Prompt Provided to Initial Reviewer AI Agent for Blood Culture Appropriateness Classification AI agents were guided by structured inclusion and exclusion criteria to assess blood culture appropriateness. Prompts included clinical definitions, required supporting evidence, and explicit instructions to avoid assumptions or external reasoning beyond the documentation in the clinical note. Agents were also asked to provide quoted justification for their classifications. The criteria were adapted from the Johns Hopkins Prevention Epicenter Blood Culture Stewardship Collaborative algorithm and based on: Fabre V, Sharara SL, Salinas AB, Carroll KC, Desai S, Cosgrove SE. Does This Patient Need Blood Cultures? A Scoping Review of Indications for Blood Cultures in Adult Nonneutropenic Inpatients. Clin Infect Dis. 2020; PMID: 31942949. Prompt Provided to Double Checker AI Agent for Verification of Blood Culture Appropriateness Assessment

**Figure:**
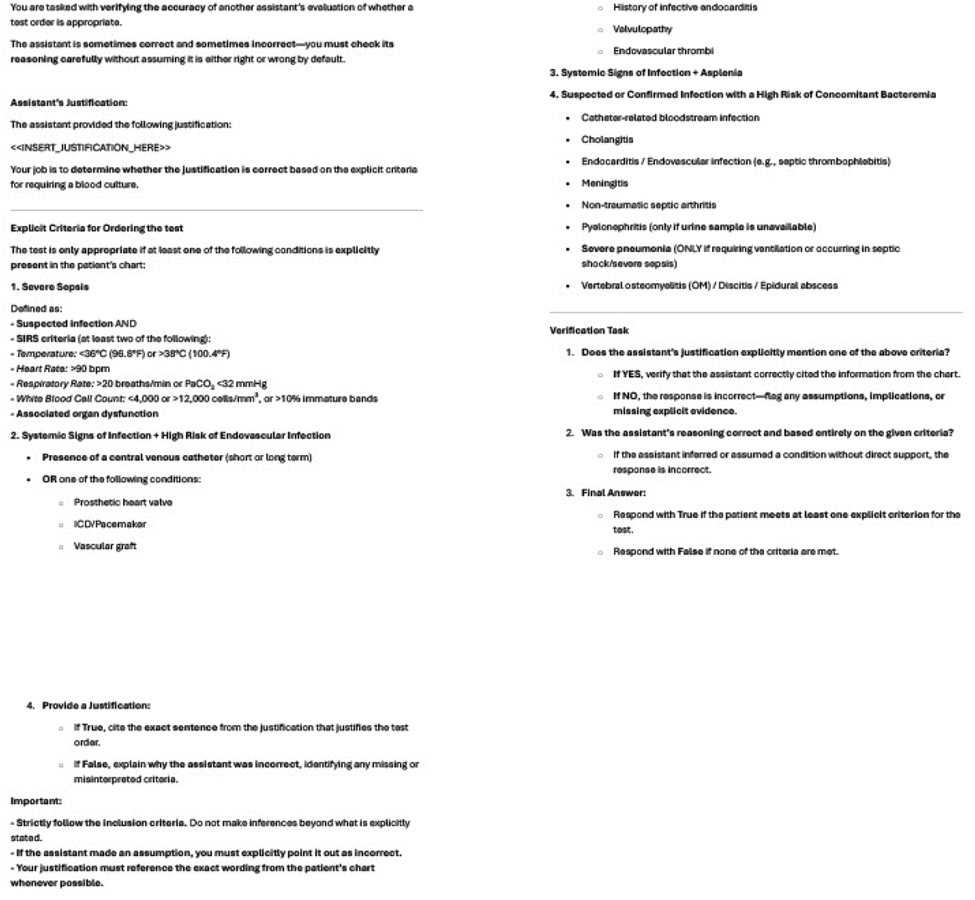
The second AI agent was tasked with verifying the initial reviewer’s justification against explicit inclusion criteria for blood culture ordering. The prompt instructed the agent to identify whether the justification explicitly met at least one inclusion criterion and to flag assumptions or unsupported reasoning. Final classifications required quoting or rejecting specific evidence from the patient’s chart.

**Methods:**

Stanford University deployed secure LLMs with direct access to electronic medical records. Using these, we developed two artificial intelligence (AI) agents—task-specific models designed to audit blood culture order appropriateness based on previously published criteria. We applied the agents to a random sample of 105 blood culture orders previously audited by an infectious diseases provider between May and December 2024. After excluding repeat orders within 48 hours, 67 unique cases remained (31 appropriate, 36 not). Each case included all assessment and plan notes from admission to blood culture collection (range: 1–500 notes). The initial reviewer agent (gpt-4o-mini; OpenAI) scanned the notes for any mention of appropriateness or non-appropriateness criteria. A second, more powerful double-checker agent (o1-mini; OpenAI) then reviewed and, if necessary, corrected the initial classification.

**Figure:**
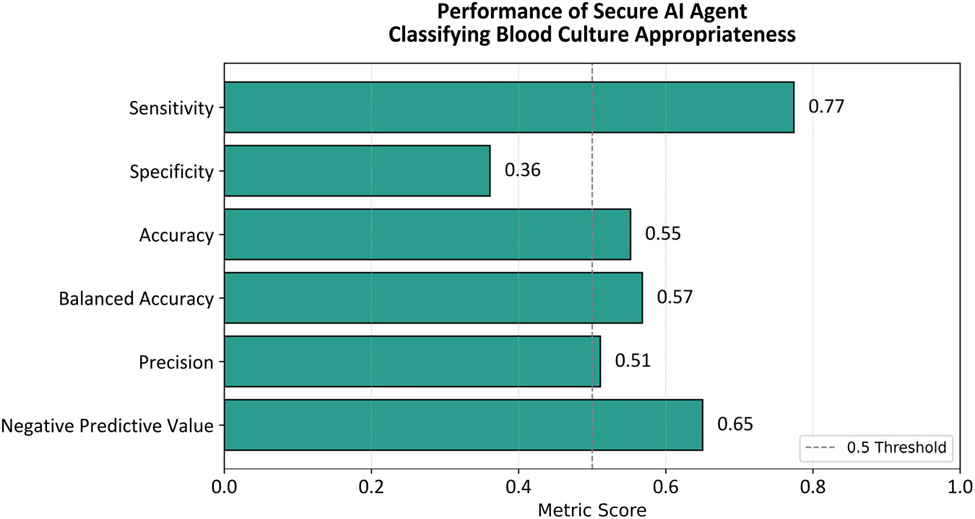
Performance Metrics of Secure AI Agent Classifying Blood Culture Appropriateness — Northern California, 2025 The AI agent's performance is summarized across six standard classification metrics. While sensitivity and negative predictive value were relatively high, specificity and precision were lower, reflecting a tendency to overflag orders as appropriate. The 0.5 reference threshold is marked for interpretability.

**Figure:**
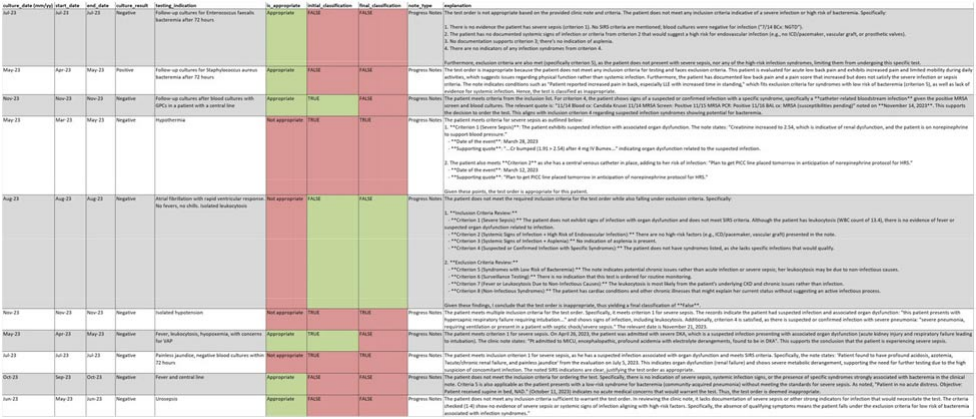
Case-Level Examples of Blood Culture Orders Reviewed by AI Agents and Adjudicated for Appropriateness Each row represents a single blood culture order with corresponding adjudication status, AI agent classifications, and rationale. Green indicates an appropriate order; red indicates an inappropriate order. AI explanations were based on clinical notes available prior to blood culture collection. Discrepancies between human adjudication and AI classification are highlighted in the initial and final classification columns.

**Results:**

Overall performance of the AI agents was modest, with a balanced accuracy of 0.568, sensitivity of 0.774, and specificity of 0.361. The agents frequently over-flagged blood culture orders as appropriate, demonstrating a tendency to recommend blood cultures in a broad range of cases. This likely reflects a known LLM behavior, *sycophancy*, where the model aligns with the reasoning presented in the clinical notes, such as agreeing with the care team’s suspicion of sepsis, even when objective criteria were not met. Notably, the “severe sepsis/septic shock” criterion was the most common justification given by the AI agents for classifying orders as appropriate.

**Conclusion:**

The AI agents demonstrated limited performance in adjudicating blood culture appropriateness. Their decisions were largely influenced by sycophantic bias and the presence of the word *sepsis* in the notes. Their utility in medical classification tasks may be best suited for initial screening rather than clinical recommendations.

**Disclosures:**

All Authors: No reported disclosures

